# Dr. Milligan's Second Edition of Celsus

**Published:** 1831

**Authors:** 


DR. MILLIGAN'S SECOND EDITION OF CELSUS.

THIS second edition of Celsus has had the advantage of being reprinted, with the greatest attention to accuracy, by Dr. Milligan, from his former edition, which was generally pronounced immaculate by some of the most classical of our medical reviewers. But the editor has not confined his attention to the sole object of giving Celsus again to the world, with a high degree of literal precision; he has made many additions and improvements, several of which must greatly enhance the value of the work to the reader; and we are happy to state that he has been enabled to effect so much without any addition to the price of the work. Thus there is a Delphini index verborum, in quadruple columns of nearly ten densely printed sheets, by which this distinguished classic is now put upon the same footing with other ancient authors so published; and must prove a valuable auxiliary to every scholar who would study the elegant and highly idiomatic language of Celsus; nor less so to the man of the profession who would acquire the power of delivering his ideas to the world in the dialect of the Augustan age. There is a synopsis of the different articles and instruments employed by Celsus, illustrated with their English synonymes, an improvement which renders the work intelligible to readers not familiar with natural history or botany. The authorities, documents, and principles upon which the relations of ancient measures to each other and to modern measures are established, have been given complete in the present edition, and the old index Capitum, which was allowed by all to be useless, has been instructively converted into an analytical table of contents, which affords to a glance of the eye a distinct view of the whole plan and connexion of the work of Celsus.

To the catalogue of authors mentioned by Celsus, has been added the periods at which they flourished, the books in which such dates are to be found, being in very few hands. Nothing useful has been retrenched in order to make way for these improvements; and we think, therefore, that the editor may rationally anticipate to his present labours a continuance of that partiality which the public has evinced towards his first edition.

---

